# Association of frontal plane knee alignment with foot posture in patients with medial knee osteoarthritis

**DOI:** 10.1186/s12891-017-1588-z

**Published:** 2017-06-07

**Authors:** Hiroshi Ohi, Hirotaka Iijima, Tomoki Aoyama, Eishi Kaneda, Kazuko Ohi, Kaoru Abe

**Affiliations:** 1 Graduate School of Health and Welfare, Niigata University of Health and Welfare, Niigata, Japan; 2 Ohi Manufacturing Co., Ltd., Kyoto, Japan; 3 Department of Physical Therapy, Human Health Sciences, Graduate School of Medicine, Kyoto University, Kyoto, Japan; 4 Department of System Design Engineering, Keio University, Yokohama, Japan; 5 Japan Society for the Promotion of Science, Tokyo, Japan; 6 Nozomi Orthopaedic Clinic, Hiroshima, Japan

**Keywords:** Osteoarthritis, Foot posture, Anatomical alignment, Hallux valgus

## Abstract

**Background:**

To examine the association of radiographic frontal plane knee alignment with three-dimensional foot posture in patients with medial knee osteoarthritis (OA).

**Methods:**

Participants in orthopedic clinics with Kellgren/Lawrence (K/L) grade ≥1 (88 patients and 88 knees; age, 61–91 years; 65.9% female) were enrolled. An anteroposterior radiographic view was used to assess the anatomical axis angle (AAA) after subtracting a sex-specific correction factor. The three-dimensional foot posture was also evaluated.

**Results:**

Multiple regression analyses showed that increased corrected AAA (i.e., valgus direction) was independently associated with a decrease in the hallux valgus angle (regression coefficient: −0.40 per degree, 95% confidence interval [CI]: −0.72, −0.09; *P* = 0.013) and increase in the pronation angle of the calcaneus relative to floor (regression coefficient: 0.33 per degree, 95% CI: 0.10, 0.56; *P* = 0.005) adjusted for age, sex, and body mass index. The relationship between the corrected AAA and hallux valgus angle strengthened (regression coefficient: −0.60 per degree, 95% CI: −1.08, −0.13; *P* = 0.014) in varus-aligned knees examined separately (63 knees). The other foot postures (navicular height, navicular height/foot length, and rearfoot angle) were not significantly associated with corrected AAA.

**Conclusions:**

Radiographic frontal plane knee alignment was associated with hallux valgus angle and calcaneus angle relative to the floor in patients with medial knee OA, particularly in varus-aligned knees. These results indicate a connection between altered frontal knee alignment and foot posture, which may be helpful in understanding the pathogenesis of altered foot posture observed in patients with knee OA.

## Background

Knee osteoarthritis (OA) is one of the leading diseases responsible for knee pain and disability [1, 2]. Progression of the disease is widely believed to result from local mechanical factors, [3] and much of the previous research has focused on local knee malalignment [4, 5]. However, people with knee OA exhibit an altered foot posture including flatfoot, pronated foot, and hallux valgus more frequently than healthy adults [6–9]. Flatfoot is associated with the presence of knee pain and medial cartilage damage in patients with knee OA [10]. Hallux valgus is associated with both knee and foot pain as well as the presence of nodal OA [11] and an increase in disability levels in women with knee OA [12]. A recent study showed that approximately 13–39% of individuals with knee OA, or at-risk of knee OA, have concurrent foot pain that adversely affects their functional status [13], knee symptoms [14], and clinical outcomes 1 year following total knee arthroplasty (TKA) [15].

The foot is thought to play an important role in knee OA from a biomechanical perspective owing to rotational coupling between the rearfoot and tibia [16, 17]. A simulated genu varum walking pattern increases the subtalar pronation moment, whereas a simulated genu valgum walking pattern increases the subtalar supination moment [18]. Furthermore, accumulated evidence shows that realignment of the knee following TKA results in changes in hindfoot alignment [19–21] and foot kinematics during gait [22]. Although this evidence suggests the existence of a biomechanical link between altered frontal plane knee alignment and altered foot posture in patients with knee OA, we are unaware of any studies that have investigated the direct relationship of frontal plane knee alignment and foot posture, including forefoot, midfoot, and rearfoot postures, within this population. Since some patients with knee OA persistent hindfoot pain after TKA, [23] this knowledge would be helpful to understand the altered foot morphology related to knee OA pathology and to provide the basis for establishing the pathogenesis of concurrent foot pain in patients with knee OA. This may be important, given that patients with knee OA already have knee pain and disability, [1, 24] and the coexistence of altered foot posture may aggravate these symptoms.

Currently, the gold standard measure of knee alignment is the mechanical axis (or hip-knee-ankle) angle. However, it requires full-limb radiographs and the expertise of a radiology technologist to overcome the inherent technical difficulty. Alternatively, the measure of the anatomical axis angle (AAA) has been validated as a comparable measure to the full-limb radiographs, [25, 26] has been used for evaluating knee alignment, is less technically difficult and less costly. Thus, this study aimed to examine the association of radiographic frontal plane knee alignment measured as AAA with three-dimensional foot posture in patients with medial knee OA. General hypothesis was that increased frontal plane knee varus alignment independently associated with increases in the hallux valgus angle and rearfoot valgus angle and decreases in medial arch (navicular) height.

## Methods

### Patients

This cross-sectional study included outpatients with knee OA diagnosed by their treating physician in community orthopedic clinics. This study is the same cohort of subjects of recently published article [27]. For recruitment, an advertisement was distributed to patients who sought conservative treatment for knee OA in January 2015.

The eligibility criteria included (i) age ≥50 years; (ii) knees with radiographic OA (i.e., Kellgren/Lawrence [K/L] [28] grade ≥1) in one or both knees, as evaluated by weight-bearing anteroposterior radiographs; and (iii) an ability to walk independently on a flat surface without any ambulatory assistive device. The exclusion criteria were (i) a history of knee surgery, (ii) inflammatory arthritis, (iii) periarticular fracture, (iv) neurological problems, or (v) lateral compartment knee OA. Lateral knee OA was defined as a knee having a K/L grade ≥1 along with joint space narrowing (JSN) >0 in the lateral compartment with JSN = 0 in the medial compartment [29, 30]. Since medial and lateral knee OA have distinct characteristics, and most knee OA in Japan is medial type, [31] lateral knee OA was excluded in this study. Because pre-radiographically defined knee OA, particularly K/L grade 1, predicts radiographic OA progression to at least grade 2, [32, 33] we included patients with K/L grades ≥1. Patients with either bilateral or unilateral knee OA were considered.

## Measurements

### Radiographic evaluation of the knee joint

The radiographic OA severity of both knees in each patient was assessed in the anteroposterior short view in the weight-bearing position by experienced examiners using the original version of the K/L grading system [28, 34]. Specifically, the K/L grade was scored as follows: 0 = normal; 1 = doubtful JSN and possible osteophyte; 2 = definite osteophyte and possible JSN; 3 = multiple osteophytes, definite JSN, and some sclerosis and possible deformity of bone ends; and 4 = large osteophyte, marked JSN, severe sclerosis, and definite deformity of bone ends. The interrater agreements for the K/L grade determination were excellent (κ: 0.84, 95% confidence interval [CI]: 0.79, 0.90).

Frontal plane knee alignment was evaluated by measurement of the AAA on an anteroposterior radiograph by a trained examiner (HI) using the Nazca software (Astrostage Inc., Tokyo, Japan). The AAA was defined as the internal angle formed by the intersection of two lines originating 10 cm from the knee joint surfaces bisecting the femur and tibia, and converging at the center of the tibial spine tips [35]. The exact distance from the knee joint surface at which the femur or tibia was to be bisected was determined on the basis of the size of the patients; a length 1.3 times that of the epicondylar line from the medial epicondyle to the lateral epicondyle was used. Measurements of the AAA were modified to better reflect mechanical alignment by subtracting a sex-specific correction factor of 3.5° for women and 6.4° for men according to Kraus; [25] throughout the text, the corrected AAA is described. Sex-specific corrections on AAA has also been shown to be important [36, 37]. Increase and decrease in corrected AAA signifies valgus and varus postures, respectively. The need for sex-specific correction has been proposed because women have more distal femoral valgus than men [38]. The knees were categorized based on corrected AAA into three groups: neutral (corrected AAA ≥179° but <182°), varus (corrected AAA <179°), and valgus (corrected AAA ≥182°) alignments. The intrarater reliability for measuring the AAA was excellent (intraclass correlation coefficient [ICC]: 0.98, 95% CI, 0.98, 0.99).

### Evaluation of the three-dimensional foot posture and rearfoot angle

**Fig. 1:**
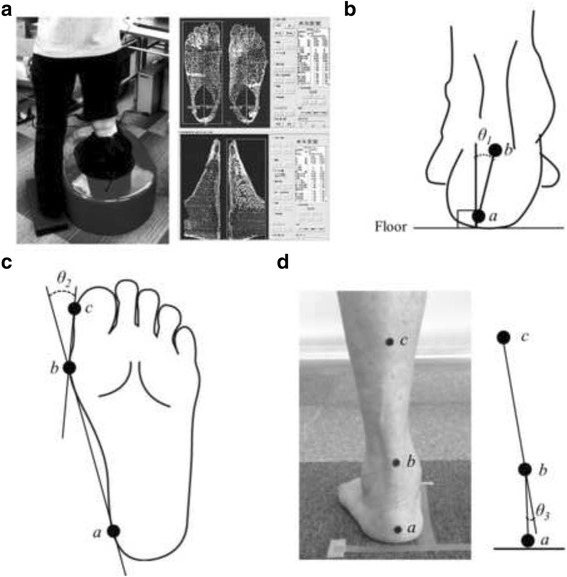
Evaluation of foot posture and rearfoot angle. **a** Foot-scanning system to evaluate three-dimensional foot posture. **b** Measurement of calcaneus angle relative to the floor (*θ1*), which was defined as the angle formed by a line joining the bottom of the calcaneal tuberosity (*a*) with the enthesis of the Achilles tendon (*b*) and a line perpendicular to floor. **c**, Measurement of the hallux valgus angle (*θ2*). The hallux valgus angle (*θ2*) was defined as two lines intersecting between a line connecting the medial side of the first metatarsophalangeal joint (*b*) with the medial side of the heel (*a*) and a line connecting the medial side of the first metatarsophalangeal joint (*b*) with the medial side of the hallux (*c*). **d** Measurement of rearfoot angle (*θ3*), which was defined as the angle between the bisection of the lower one-third of the leg (*bc*) and the bisection of the calcaneus (*ab*). Note that the measurement procedures of foot length and navicular height are not indicated in this figure

Three-dimensional foot posture was evaluated using a three-dimensional footprint automatic measurement apparatus (CUTE, JMS-2100CU; Dream GP Inc., Osaka, Japan) (Figure 1a). The details regarding the measurement setup are provided in the previous study [27]. This foot-scanning system is based on laser line triangulation where the measuring head moves around a single foot in an oval trajectory [39]. The laser scanner rotates around the patient’s foot and measures >30,000 points, including the ankle, instep, heel, and toes as well as the sole, recreating the patient’s foot shape precisely. This scanning system has a high accuracy for measurements of foot posture; the measurement error of foot length is −0.27 to 0.36 mm (accuracy within ± 0.2%) and that of foot width is 0.51–1.22 mm (accuracy within ± 0.5%) [39].

Before each capture session, the patients stood on bare feet with feet shoulder-width apart as straight as possible with little movement, and black round seal markers, which correspond to three anatomical landmarks to detect foot alignment, were attached to skin. This placed 50% of their body weight on the foot being assessed. The anatomical landmarks were the (i) navicular tuberosity, (ii) bottom of the calcaneal tuberosity, and (iii) enthesis of the Achilles tendon. The markers were attached by an experienced prosthetist and orthotist (HO) who had >15 years of clinical experience in the orthopaedic field, with no knowledge of each patient’s clinical status, such as OA severity. The measurement procedure consisted of consecutive right and left foot scanning, with each taking approximately 13 s.

After the measurement step, the following foot postures were automatically calculated by the system: calcaneus angle relative to the floor, foot length, hallux valgus angle, navicular height, and navicular height/foot length. The calcaneus angle relative to the floor was defined as the angle formed by a line joining the bottom of the calcaneal tuberosity with the enthesis of the Achilles tendon and a line perpendicular to floor (Figure 1B). The foot-scanning system defined the floor line and automatically calculated the line perpendicular to the floor. Increases and decreases in calcaneus angle relative to the floor indicate pronation and supination, respectively. Throughout the manuscript, “pronation/supination” indicates posture on a single frontal plane. As hallux valgus is often found in patients with knee OA, [9] foot length was defined as the distance from the most posterior portion of the calcaneus to the end of the second toe. The hallux valgus angle was defined as two lines intersecting between a line connecting the medial side of the first metatarsophalangeal joint with the medial side of the heel and a line connecting the medial side of the first metatarsophalangeal joint with the medial side of the hallux (Figure 1C). Hallux valgus was defined as a hallux valgus angle ≥20° [40]. Navicular height was defined as the distance from the floor to the navicular tuberosity.

After evaluation of the three-dimensional foot posture, the rearfoot (calcaneus) angle relative to the lower leg in a relaxed standing position was also evaluated to provide information about the subtalar joint. Before each evaluation, the patients stand as straight as possible as in the evaluation of the three-dimensional foot posture, and an additional black round seal marker was attached onto the skin of the midline of the lower one-third of the leg. The lower leg and rearfoot images from the back view were recorded using a digital camera (IXY DIGITAL 22015; Canon Inc., Tokyo, Japan). Two trained examiners measured the rearfoot angle as the angle between the bisection of the lower one-third of the leg and the bisection of the calcaneus, [41] referring to the three anatomical landmarks mentioned above (i.e., midline of lower one-third of the leg, enthesis of the Achilles tendon, and bottom of the calcaneal tuberosity) using ImageJ software. The interrater reliability for measuring the rearfoot angle was excellent (ICC: 0.92, 95% CI: 0.89, 0.94).

### Assessment of covariates

Data on age, sex, and height were self-reported by the patients. Weight was measured on a scale, with the participants wearing their clothes but without their shoes. Body mass index (BMI) was calculated by dividing the weight by the square of height.

### Statistical analysis

To minimize any bias produced by similarities between the right and left knees of the same patients, [42] only one knee per patient was analyzed, the “index knee.” The index knee was defined as the more painful knee in the present or past. If patients felt that their knees were equally painful, the index knee was randomly selected using computer-generated block randomization.

To clarify the associations between frontal knee alignment and three-dimensional foot posture when standing, regression coefficients (betas) and their 95% CIs were calculated using two models (a crude model and adjusted model) of multiple regression analyses. In the crude model, each foot posture (hallux valgus angle, navicular height, navicular height/foot length, calcaneus angle relative to floor, and rearfoot angle) was included as a dependent variable and the corrected AAA (continuous) was included as a predictor. The multiple regression analyses were done first with a crude model, which was then adjusted for age (continuous), sex (0: male, 1: female), and BMI (continuous) for adjusted model. These covariates were chosen because they might be associated with the alterations of the frontal plane knee alignment and foot posture and not on the causal pathway [11, 43, 44].

Sensitivity analyses were performed to assess whether the relationship between the corrected AAA and foot posture is influenced by the subsample of knees with K/L grade ≥2. Furthermore, to address the possibility that the relationship of the corrected AAA with foot posture differs when patients are subdivided into those with (corrected AAA <179°) and without (corrected AAA ≥179°) varus alignment, additional multiple regression analyses were performed in each subgroup separately. In these analyses, we replicated the same multiple logistic regression analyses as mentioned earlier. Data analyses were performed with JMP version 11 (SAS Institute, Cary, NC, USA). *P*-values <0.05 were considered statistically significant.

## Results

**Fig. 2:**
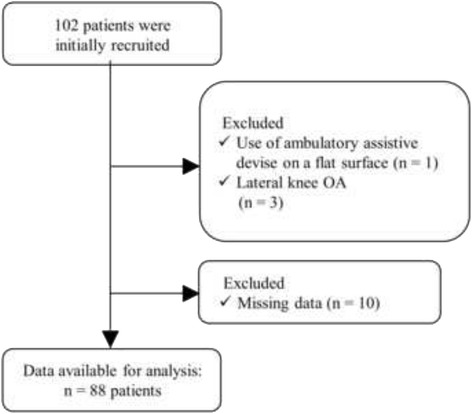
Flow chart describing the distribution of study patients

A flow chart describing the distribution of study patients is shown in Figure 2. In this study, 102 patients were initially enrolled. However, 4 patients were excluded (owing to use of an ambulatory assistive device on a flat surface and lateral knee OA), and 10 patients were also excluded owing to invalid data (missing data) of outcome variables; the remaining 88 patients with 88 index knees of K/L grade ≥1 (86.3% of the initial cohort) were included in the final analysis. Table 1 shows the person-level and knee-level characteristics of the study patients.

**Table 1:** Demographic characteristics, knee alignment, and three-dimensional foot posture of the study patients (*n* = 88 patients, 88 knees)

Person-level characteristics	
Age, years	74.8 ± 7.58
Female, no. (%)	58 (65.9)
Height, m	1.56 ± 7.89
Weight, kg	59.2 ± 10.5
Body mass index, kg/m^2^	24.3 ± 3.54
Knee-level characteristics	
Corrected AAA, degree	176.6 ± 4.77
Tibiofemoral joint alignment, no. (%)	
Neutral (corrected AAA ≥179 degrees but <182 degrees)	20 (22.7)
Valgus (corrected AAA ≥182 degrees)	5 (5.7)
Varus (corrected AAA <179 degrees)	63 (71.6)
Tibiofemoral joint K/L grade, no. (%)	
Grade 1	30 (34.1)
Grade 2	35 (39.8)
Grade 3	14 (15.9)
Grade 4	9 (10.2)
Hallux valgus angle, degree	13.6 ± 7.22
Presence of hallux valgus, no. (%)^a^	11 (12.5)
Navicular height, mm	30.1 ± 6.75
Navicular height/foot length, %^b^	12.7 ± 2.75
Calcaneus angle relative to floor, degree^c^	1.35 ± 5.09
Rear foot angle, degree^d^	6.01 ± 3.76

Values are mean ± standard deviation unless indicated otherwise. AAA = anatomical axis angle; K/L = Kellgren/Lawrence

^a^Presence of hallux valgus is defined as the hallux valgus angle ≥20°

^b^Navicular height/foot length is calculated by the navicular height divided by the foot length

^c^A positive value indicates pronation direction of the calcaneus

^d^A positive value indicates valgus direction of the rearfoot

### Corrected AAA was independently associated with the hallux valgus and calcaneus angles

The results of the multiple regression analyses characterizing the association between the corrected AAA and three-dimensional foot posture are shown in Table 2. An increased corrected AAA (i.e., valgus direction) was significantly associated with a decrease in the hallux valgus angle (beta: −0.40 per degree, 95% CI: −0.71, −0.09; R^2^ = 0.07; *P* = 0.012) even when adjusted for age, sex, and BMI (beta: −0.40 per degree, 95% CI: −0.72, −0.09; R^2^ = 0.14; *P* = 0.013). These relationships were still significant even when the calcaneus angle and navicular height were further included as predictors (data not shown).

**Table 2:** Regression coefficient from multiple regression analysis, characterizing the association between increased corrected AAA (per degree) and three-dimensional foot posture (*n* = 88 knees)

Dependent variables	Beta (95% CI) of corrected AAA, per degree*	
	Crude model	Adjusted model
Hallux valgus angle	−0.40 (−0.71, −0.09)*	−0.40 (−0.72, −0.09)*
Navicular height	−0.02 (−0.32, 0.29)	0.08 (−0.21, 0.38)
Navicular height/foot length	−0.002 (−0.13, 0.12)	0.03 (−0.10, 0.15)
Calcaneus angle relative to floor	0.31 (0.09, 0.53)**	0.33 (0.10, 0.56)**
Rearfoot angle	−0.10 (−0.27, 0.06)	−0.09 (−0.26, 0.08)

95% CI = 95% confidence interval; AAA = anatomical axis angle

The value of regression of coefficient (beta) for each dependent variable is described per degree of AAA (continuous) to indicate the predictive ability using 2 cumulative models of multiple regression analyses

Adjusted model includes values derived from multiple regression analysis with age, (continuous), sex (0: male, 1: female), and body mass index (continuous) entered simultaneously (one-step model) as predictors

*P < 0.05, **P < 0.01. Bold indicates a statistically significant result

An increased corrected AAA was significantly associated with increases (i.e., pronation direction) in the calcaneus angle relative to the floor (beta: 0.31 per degree, 95% CI: 0.09, 0.53; R^2^ = 0.08; *P* = 0.006) even when adjusted for age, sex, and BMI (beta: 0.33 per degree, 95% CI: 0.10, 0.56; R^2^ = 0.10; *P* = 0.005). However, the other foot postures (i.e., navicular height and navicular height/foot length) were not significantly associated with corrected AAA with the numbers available (navicular height, beta: 0.08 per degree, 95% CI: −0.21, 0.38; R^2^ = 0.16; *P* = 0.566; navicular height/foot length, beta: 0.03 per degree, 95% CI: −0.10, 0.15; R^2^ = 0.07; *P* = 0.688). Also, an increased corrected AAA was not significantly associated with rearfoot angle with the numbers available (beta: −0.09 per degree, 95% CI: −0.26, 0.08; R^2^ = 0.07; *P* = 0.290).

Sensitivity analysis showed that these relationships were comparable; there were significant associations between the increased corrected AAA and decrease in the hallux valgus angle (beta: −0.46 per degree, 95% CI: −0.83, −0.08; R^2^ = 0.23; *P* = 0.018) as well as calcaneus angle relative to the floor (beta: 0.33 per degree, 95% CI: 0.06, 0.60; R^2^ = 0.11; *P* = 0.015) adjusted for age, sex, and BMI in knees with K/L grade ≥2 (*n* = 58).

### The association of corrected AAA with the hallux valgus and calcaneus angles became stronger in varus-aligned knees

When only the varus knees (*n* = 63) were analyzed, the association between the increased corrected AAA and the decrease in hallux valgus angle became strong adjusted for age, sex, and BMI (beta: −0.60 per degree, 95% CI: −1.08, −0.13; R^2^ = 0.17; *P* = 0.014). The association between the increased corrected AAA and the increase in calcaneus angle relative to floor also became stronger when adjusted for age, sex, and BMI (beta: 0.45 per degree, 95% CI: 0.13, 0.76; R^2^ = 0.14; *P* = 0.006). However, the associations between the corrected AAA and the other foot postures as well as the rearfoot angle in varus-aligned knees were similar (data not shown) compared to values shown in Table 2. Moreover, when only the non-varus knees (*n* = 25) were analyzed, the association of the corrected AAA with the hallux valgus was not significant adjusted for age, sex, and BMI with the numbers available (beta: −0.35 per degree, 95% CI: −2.12, 1.42; R^2^ = 0.16; *P* = 0.682).

## Discussion

Alterations of foot morphology in patients with knee OA are associated with knee pain, disability, and cartilage damage [10–12]. Although several studies suggest the existence of a biomechanical connection between frontal knee alignment and rearfoot angle, [16–22] the relationship of frontal plane alignment and foot posture of patients with knee OA has not been fully elucidated. The current study first revealed that an increased corrected AAA (i.e., valgus direction) was independently associated with decreases in the hallux valgus angle and decreases in the calcaneus angle relative to the floor. Furthermore, increased corrected AAA was more closely associated with decreases in the hallux valgus angle as well as increases in the calcaneus angle relative to floor in varus-aligned knee, examined separately. However, there were no significant associations in non-varus-aligned knee. Although variance in the multiple regression model was relatively small (R^2^ < 0.2), even on the association between corrected AAA and hallux valgus, our data may suggest that altered frontal knee alignment is associated with altered foot posture, particularly the hallux valgus angle in varus-aligned knee.

Hallux valgus is a common deformity with a wide prevalence rate ranging from 23 to 64% [9, 11, 40]. Lower prevalence rate of hallux valgus in this study (12.5%) could be attributed to the definitions of hallux valgus used; previous studies used physical examination, [9] self-reported questionnaire, [11] or foot radiograph in the weight-bearing position [40]. Hallux valgus is associated with a higher risk of foot and knee pain, [11] increased disability in women with knee OA, [12] and was linked to the progression of knee OA in a case study [45]. Although nonsurgical care is the first option for patients who have hallux valgus deformity, [46] many patients require surgery, [47] which increases healthcare costs [48]. The current study first revealed that a frontal plane knee alignment was independently associated with the hallux valgus angle, which is comparable to a subsample of knees with a K/L grade ≥2, and this relationship became stronger in varus-aligned knees. Although the current study cannot elucidate the mechanism to explain the relationship between knee alignment and hallux valgus angles, several studies showed that patients with hallux valgus have an altered foot pressure pattern during gait in patients with medial knee OA [49–52]. The AAA was correlated with the coronal location of the center of pressure, [51] and realignment of the knee following TKA results in a foot pressure pattern, particularly in the hallux [53]. These results indicate a correlation between altered frontal plane knee alignment and altered pressure on the hallux.

The altered rearfoot posture of pronation in patients with knee OA has been proposed to be a compensatory response to the varus alignment to allow the foot to be plantigrade [54]. However, contrary to our initial hypothesis, we found that increased varus knee alignment (decreased corrected AAA) was associated with an increase in the supination angle of the calcaneus relative to the floor. This discrepancy is possibly attributed to a reduced compensatory capacity of the ankle/subtalar joint complex or a restricted range of motion in subtalar pronation. Hindfoot varus deformity exists in 30% of cases of end-stage varus knee OA, [21, 55] and hindfoot varus is believed to occur as a result of the loss of this compensatory capacity of the hindfoot. A prospective study to follow-up on the foot posture in patients with knee OA who had varus alignment would help address this question.

Interestingly, the relationship of the corrected AAA with the rearfoot angle, an indicator of varus or valgus alignment of the subtalar joint, was not significant when adjusted for possible confounders. On the other hand, the significant relationship of the corrected AAA with the calcaneus angle relative to the floor indicates that the calcaneus angle might be more sensitive to the altered frontal plane alignment. These discrepancies of assessment techniques for calcaneus and rearfoot angles might explain why there is no significant relationship of the rearfoot angle with altered frontal knee alignment in this study. Norton et al. showed that the relationship of the mechanical axis and the hindfoot valgus angle was stronger in severe varus alignment, but this relationship was rather diminished in patients with a milder varus alignment [20]. We also found no significant relationship between the corrected AAA and calcaneus angle relative to the floor or the rearfoot angle in the non-varus-aligned knee, supporting their finding.

We found that navicular height and navicular height/foot length, two of the midfoot alignments, were not significantly associated with the corrected AAA. Navicular height in patients with knee OA is similar to that in healthy adults, [8] even in patients diagnosed to have a pronated foot according to foot posture index and arch index [6, 56]. The association of the rearfoot with navicular drop was only moderate even in healthy adults [57]. Furthermore, we found only a weak relationship between the calcaneus angle and navicular height/foot length (data not shown), which might explain why the corrected AAA was associated only with the calcaneus angle but not navicular height in this study.

Rearfoot pronation results in an increase in loading to the first metatarsal, [58] which might contribute to the degenerative changes in the first metatarsophalangeal joint on the same side as medial knee OA [45, 59]. However, we also found that including navicular height (i.e., an indicator of medial arch) and calcaneus angle (i.e., an indicator of rearfoot alignment) in the multiple regression model as a predictor did not affect the regression coefficient of the corrected AAA on the hallux valgus angle, indicating a weak influence of the medial arch and rearfoot alignment on the association of frontal plane knee alignment and the hallux valgus angle. Nix et al. recently reviewed 37 papers to detect foot structure associated with hallux valgus and found inconsistent results regarding flat foot as a risk factor [60]. Thus, there is a possibility that systemic factors, rather than biomechanical factors, might contribute to the association of frontal plane knee alignment and the hallux valgus angle. Radiography-detected first metatarsophalangeal joint OA is associated with radiographic OA at the distal and proximal interphalangeal joint, first carpometacarpal joint, and knee joint, suggesting hallux valgus as a component of generalized OA [61].

This study has some limitations. First, this is a cross-sectional study without any sample size calculation. As such, the causal relationship of frontal plane knee alignment and foot postures cannot be inferred, and the results should be interpreted with caution because of a wide 95% CI of beta. Furthermore, without a control group, it is unclear whether these changes are the result of OA degenerative process, or merely a reflection of a normal relationship between foot posture and knee alignment. We found that significant relationships between foot posture and knee alignment were independent from K/L grade of index knee (data not shown), which counters the theory that all of these changes are the results of some OA process. Our data sets the foundation for a prospective cohort study with the aim of clarifying the pathogenesis of altered foot posture observed in patients with knee OA. Second, since study participants were limited to medial knee OA (94.3% with varus or neutral alignment), these results may not directly translate to a general population with knee OA. Third, evaluation of three-dimensional foot posture is based on laser line triangulation, which captures foot morphology [39]. This skin-based scanning system may be affected by body composition, such as BMI. In this way, radiography-based evaluation would facilitate the identification of an exact relationship between frontal plane knee alignment and foot posture. Fourth, we used short anteroposterior radiographs to assess the AAA as opposed to the accepted gold standard of using long-limb radiographs to measure the mechanical axis angle. Furthermore, our methods do not account for the transverse plane, which may have an impact on the association between knee alignment and foot postures. Given that varus alignment may need greater offset than valgus alignment on the prediction of mechanical axis from AAA, and offset depends on degree of knee deformity [36], the true mechanical axis angle considering knee rotational alignment would be helpful to understand the altered foot morphology related to knee OA pathology. Nevertheless, AAA with sex-specific correction is well correlated with the mechanical axis angle, [25, 36, 37] and it can be used even when a full-limb radiograph is not available. Furthermore, we found that other offsets such as sex-specific correction factors of 3.0° or 4.6° for women and 4.7° or 6.5° for men [36, 37] and regression analysis (mechanical axis = 0.915 × AAA + 13.895), [26] did not change the relationship between knee and foot alignments (data not shown). Fifth, this study lacks reproducibility data of black seal marker attachments on foot, which may impact the relationship between knee alignment and foot posture. Unblinding marker attachments due to observation of the patients during markers attachment is also an important limitation. However, we performed a pilot study and found good reproducibility of marker attachments on navicular tuberosity in healthy adult (data not shown). Furthermore, an experienced prosthetist and orthotist clinician performed these marker attachments without knowledge of each patient’s clinical status, which is a strength point in this study. Finally, the foot scanning system used in this study only evaluated static measurements while standing. This may explain why variance explained in the model was relatively small, particularly on the association between corrected AAA and hallux valgus. Small variance limits the ability to determine the clinical impact of relationships between knee alignment and foot posture. While standing, approximately half of the external loading applied to the talus is transmitted to the heel [58]. Measurements that capture dynamic alignment (e.g., higher loading phase onto the toe, such as in terminal stance during gait) might provide practical information about the association of altered frontal knee alignment and forefoot alignment. Indeed, patients with medial knee OA display altered hallux and midfoot kinematics, particularly in the terminal stance phase of gait compared to healthy adults [62].

## Conclusions

Corrected AAA evaluated by radiography was independently associated with the hallux valgus angle and calcaneus angle relative to the floor in patients with medial knee OA, particularly in varus-aligned knees. These results indicate the existence of a connection between altered frontal knee alignment and foot posture, which would be helpful to understand the pathogenesis of altered foot posture observed in patients with knee OA.

## Abbreviations

OA: Knee osteoarthritis
TKA: Total knee arthroplasty
AAA: Anatomical axis angle
K/L: Kellgren/Lawrence
JSN: Joint space narrowing
ICC: Intraclass correlation coefficient
BMI: Body mass index.
